# Biliary Endoprosthesis: A Prospective Analysis of Bacterial Colonization and Risk Factors for Sludge Formation

**DOI:** 10.1371/journal.pone.0110112

**Published:** 2014-10-14

**Authors:** Jochen Schneider, Alexander Hapfelmeier, Julia Fremd, Philipp Schenk, Andreas Obermeier, Rainer Burgkart, Stefanie Forkl, Susanne Feihl, Nina Wantia, Bruno Neu, Monther Bajbouj, Stefan von Delius, Roland M. Schmid, Hana Algül, Andreas Weber

**Affiliations:** 1 II. Medizinische Klinik und Poliklinik, Klinikum rechts der Isar, Technische Universität München, München, Germany; 2 Institut für Medizinische Mikrobiologie und Hygiene, Technische Universität München, München, Germany; 3 Institut für Medizinische Statistik und Epidemiologie, Technische Universität München, München, Germany; 4 Klinik für Orthopädie und Sportorthopädie, Technische Universität München, München, Germany; Rockefeller University, United States of America

## Abstract

Bacterial colonization of biliary stents is one of the driving forces behind sludge formation which may result in stent occlusion. Major focus of the study was to analyze the spectrum and number of microorganisms in relation to the indwelling time of stents and the risk factors for sludge formation. 343 stents were sonicated to optimize the bacterial release from the biofilm and identified by matrix-associated laser desorption/ionization-time of flight mass spectrometer (MALDI-TOF). 2283 bacteria were analyzed in total. The most prevalent microorganisms were *Enterococcus species (spp.)* (504;22%), followed by *Klebsiella spp.* (218;10%) and *Candida spp.* (188;8%). Colonization of the stents mainly began with aerobic gram-positive bacteria (43/49;88%) and *Candida spp*. (25/49;51%), whereas stents with an indwelling time>60 days(d) showed an almost equal colonization rate by aerobic gram-negative (176/184;96%) and aerobic gram-positive bacteria (183/184;99%) and a high proportion of anaerobes (127/184;69%). Compared to stents without sludge, more *Clostridium spp.* [(P = 0.02; Odds Ratio (OR): 2.4; 95% confidence interval (95%CI): (1.1–4.9)]) and *Staphylococcus spp*. [(P = 0.03; OR (95%CI): 4.3 (1.1–16.5)] were cultured from stents with sludge. Multivariate analysis revealed a significant relationship between the number of microorganisms [P<0.01; OR (95%CI): 1.3(1.1–1.5)], the indwelling time [P<0.01; 1–15 d vs. 20–59 d: OR (95%CI): 5.6(1.4–22), 1–15 d vs. 60–3087 d: OR (95% CI): 9.5(2.5–35.7)], the presence of sideholes [P<0.01; OR (95%CI): 3.5(1.6–7.9)] and the occurrence of sludge. Stent occlusion was found in 70/343(20%) stents. In 35% of cases, stent occlusion resulted in a cholangitis or cholestasis. In conclusion, microbial colonization of the stents changed with the indwelling time. Sludge was associated with an altered spectrum and an increasing number of microorganisms, a long indwelling time and the presence of sideholes. Interestingly, stent occlusion did not necessarily lead to a symptomatic biliary obstruction.

## Introduction

Endoscopic stent therapy is a well-established therapeutic approach in patients with biliary obstructive diseases [1]–[3]. However, stent occlusion represents a common complication in patients undergoing stent therapy, which can result in cholestasis or cholangitis [4], [5]. The risk for stent occlusion increases with the indwelling time of the stent [6]. According to literature, median stent patency ranges between 80 and 126 days [7], [8]. Therefore, stent exchanges at an interval of 3 months are routinely performed in most endoscopic centres to avoid stent occlusion [9]. Stent occlusion is mainly caused by sludge [9], [10]. Sludge consists of a mixture of microorganisms and other compounds like calcium bilirubinate, calcium palmitate, plant fibres or proteins [11]–[13]. In vitro-studies suggest that bacteria adhesion is the driving force for sludge development [10], [14]. Leung et al. [10] perfused biliary stents with either bacterially contaminated or sterile bile fluid. Scan via electron microscopy demonstrated a dense layer of bacteria and amorphous material on the surface of the stents, which were perfused with bacterially contaminated bile. In contrast, this phenomenon was not observed on the surface of the stents, exposed to sterile bile only. The authors concluded that the initiation of biliary sludge formation on biliary stents requires vital bacteria. Furthermore, any catheter embedded in the human organism constitutes a foreign matter, influencing the local bacterial flora [15], [16]. In a previous retrospective study [16], we could show that the incidences of *Enterococcus spp.* and non-fermenting bacteria were significantly higher in cholangitis episodes with biliary endoprosthesis compared to cholangitis episodes without biliary endoprosthesis. Major focus of the study was to analyze the spectrum and the number of biliary microorganisms on the surface of stents in relation to sludge, the indwelling time and the presence of sideholes on the stent surface. Furthermore, stent patency over time as well as the rate of symptomatic stent occlusions were assessed. To optimize bacterial release from the biofilm, biliary stents were exposed to low frequency ultrasound.

## Patients and Methods

### Study population

From November 2012 to December 2013, 130 patients with an elective or emergency stent exchange were consecutively included into the study. 6 patients rejected their participation to the study or could not be cleared up. Stent exchange was conducted at the II. Medizinische Klinik und Poliklinik, Klinikum rechts der Isar, Technische Universität München.

### Ethics Statement

The study was approved by the Ethics Committee, Klinikum rechts der Isar, Technische Universität München, which operates according to the Declaration of Helsinki. Written consent was obtained from most participants of the study. In a few patients, it was not possible to get a written consent and therefore oral consent was considered as sufficient basis for inclusion into the study provided that patients were cognitively able to give oral consent. All participants were fully informed about the benefits and risks of the study.

### Interventional procedure

Initial Endoscopic retrograde cholangiography (ERC): ERC was performed with a standard videoduodenoscope (TJF-160 VR). During the first ERC selective bile duct cannulation was conducted using a papillotome and a Terumo guide. In case of difficult bile duct cannulation precut techniques such as transpancreatic precut sphincterotomy or needle knife precut sphincterotomy were used. Thereafter, cholangiograms were performed by injection of contrast fluid into the bile duct. Subsequently, in most patients endoscopic sphincterotomy (EST) was carried out after placement of a stiff guide wire (e.g. Teflon guide wire). After EST, stones (or biliary sludge) were removed by using a basket or stone balloon; and strictures were dilated with a bougie or stricture balloon. In cases of biliary strictures or incomplete stone removal, (a) polyethylene stent(s) was (were) inserted. The caliber of the inserted stents varied between 7F and 11.5F.

Stent exchange and stent removal: First, the position of the indwelling biliary stent was documented with an abdominal x-ray. Thereafter, stent(s) was (were) extracted either through the working channel of the videoduodenoscope or by complete removal of the videoduodenoscope. Subsequently, a 6F ERCP catheter was inserted into the biliary tract and contrast fluid was injected. The morphological situation was (re-)evaluated by comparing previous and current cholangiograms. Depending on that, either a new polyethylene stent was inserted or stent therapy was ended. The caliber of the subsequent stents also varied between 7F and 11.5F.

### Stent characteristics and preparation

All extracted stents were made of polyethylene (Peter Pflugbeil, GmbH, Germany; Cook Incorporation, Ireland). Scanning electron microscope analysis showed similar surface conditions. To provide good preanalytic conditions, extracted stents were immediately transported to the institute of microbiology and directly prepared according to a standardized protocol: In order to minimize the risk of contamination, 1.5 centimeter of the proximal and distal end of the stent were removed using sterile scalpel and the outer surface of the stent was wiped off by a sterile compress soaked with 70% ethanol. Subsequently, the stent was opened longitudinally using a sterile scalpel and the inside of the stent assessed. The presence of sideholes was documented except for the sideholes situated at the truncated terminal ends.

### Sonication process

The prepared stent was put into an autoclaved container (Lock&Lock- container, Bandelin, Germany) and completely covered with 60 milliliters of Ringer's solution. To planktonize the microorganisms in the biofilm on the surface of the stent, the stent was vortexed for 30 seconds and subsequently exposed to low frequency (40 kHz) ultrasound for 60 seconds. The sonication process was performed in a specially for microbiological analysis designed ultrasound bath (BactoSonic, Bandelin, Germany). After the sonication process, the container was vortexed again for 30 seconds.

### Microbiological analysis

20 milliliters of the sonication fluid was centrifuged at 3000 G for 10 minutes. The supernatant was discarded, the sediment was cultivated on aerobic and anaerobic agar plates (Columbia sheep blood agar, chocolate agar, McConkey agar, Schädler anaerobic agar, Schädler KV anaerobic agar, and Sabouroud agar) and incubated in aerobic and anaerobic atmosphere at 37°C for 48 hours. Identification was conducted by matrix-associated laser desorption/ionization-time of flight mass spectrometer (MALDI-TOF, Bruker Corporation, Billerica, U.S.A.)

### Definitions of stent occlusion and sludge formation

Sludge formation was qualitatively assessed. If the sonication fluid turned after the sonication process into a yellow-brownish color, the stent was considered as sludge positive. The sonication process was standardized, using 60 ml Ringer's solution. Encrusted sludge completely narrowing the stent lumen was defined as stent occlusion.

### Statistical analysis

Statistical analysis was performed by SPSS (Version 22.0, IBM). Observations taken from several stents of the same patient were assumed to be independent. The distribution of quantitative and qualitative data is presented as median (range) or absolute and relative frequencies, respectively. Pearson's Chi-squared Test and Fisher's exact Test were used to investigate the relation of the spectrum of microorganisms to the categorized indwelling time and the occurrence of sludge, depending on the cell counts of corresponding contingency tables. In addition to this univariate analysis of potential risk factors, a multivariate analysis was performed by logistic regression. Risk factors that showed statistical significance in univariate analysis were transferred to multivariate analysis. All tests were performed on a two-sided 5% significance level.

## Results

### Patient and stent characteristics

343 biliary stents from 130 patients (77 male, 53 female) with 234 treatment episodes were analyzed. The underlying causes for stent therapy are illustrated in Table 1. Stents were extracted after a median of 70 d (1 d – 3087 d). 184 biliary stents displayed sideholes on the entire surface of the stent (presence of sideholes was not documented in one stent). Sludge was detected in 149 of 343 extracted stents. In 70 of 343 biliary stents, stent occlusion was found.

**Table 1:** Baseline characteristics.

Number of patients	130
Median age in years (range)	67(22–91)
Male	77
Reason for stent therapy	
Malignant genesis:	45
-Cholangiocarcinoma	20
-Pancreas cancer	14
-Hepatocellular carcinoma	1
-Liver metastases with intrahepatic biliary obstruction:	
-Breast cancer	1
-Colorectal cancer	6
-Gastric cancer	1
-Malignant melanoma	1
-Lymphoma	1
Benign genesis:	80
-Anastomotic stricture after liver transplantation	7
-Biliary leakage after liver transplantation	4
-Chronic pancreatitis with extrahepatic bile duct obstruction	4
-Biliary stricture after cholecystectomy	8
-Adenoma of the papilla vateri	1
-Insufficiency of the ductus cysticus after cholecystectomy	1
-Radiation induced biliary stricture	2
-Incomplete removal of biliary stones	47
-Primary sclerosing cholangitis	2
-Secondary sclerosing cholangitis	2
-Biloma	2
Idiopathic biliary stricture:	5
Total number of biliary stents	343
Number of biliary stents with sludge	149
Number of biliary stent with occlusion	70
Median duration of indwelling time in days (range)	70 (1–3087)
Total number of treatment episodes with stent extraction	234
Number of treatment episodes with multi-stenting	90
Number of elective stent extractions	197

### Microbiological analysis

Overall, 2283 microorganisms from 343 stents were isolated (Table 2), including 914(40%) aerobic gram-positive bacteria, 771(34%) aerobic gram-negative bacteria, 409(18%) anaerobic bacteria and 188(8%) *Candida spp.*. In total, 56 genera with 185 different species were identified.

**Table 2:** Spectrum of microorganisms isolated from the biliary stents.

	Total number of microorganisms	Number of stents isolated from
Number of stents		343
Total number of microorganisms	2283	
Median number of microorganisms (range)		7(0–14)
Aerobic gram-positive bacteria	914; 40%	332; 97%
Enterococcus spp.	504; 22%	297; 87%
Enterococcus faecium	126; 6%	126; 37%
Enterococcus faecalis	223; 10%	223; 65%
Other Enterococcus spp.	155; 7%	133; 39%
Streptococcus spp.	213; 9%	170; 50%
Streptococcus viridans group	211; 9%	169; 49%
Streptococcus (beta-haemolytic)	2; <1%	2; 1%
Staphylococcus spp.	39; 2%	37; 11%
Staphylococcus aureus	12; 1%	12; 3%
Staphylococcus (coagulase-negative)	27; 1%	27; 8%
Other aerobic gram-positive bacteria	158; 7%	117; 34%
Aerobic gram-negative bacteria	771; 34%	297; 87%
Enterobacteriaceae	665; 29%	279; 81%
Escherichia spp.	182; 8%	170; 50%
Klebsiella spp.	218; 10%	180; 52%
Citrobacter spp.	77; 3%	61; 18%
Proteus spp.	39; 2%	39; 11%
Morganella spp.	17; 1%	17; 5%
Enterobacter spp.	112; 5%	98; 29%
Serratia spp.	1; <1%	1; <1%
Other Enterobacteriaceae	19; 1%	19; 6%
Non-fermenting bacteria	36; 2%	36; 10%
Pseudomonas spp.	29; 1%	29; 8%
Stenotrophomonas spp.	3; <1%	3; 1%
Acinetobacter spp.	3; <1%	3; 1%
Other non-fermenting bacteria	1; <1%	1; <1%
Other aerobic gram-negative bacteria	70; 3%	58; 17%
Anaerobes	409; 18%	212; 62%
Bacteroides spp.	64; 3%	50; 15%
Prevotella spp.	149; 7%	123; 36%
Fusobacterium spp.	19; 1%	19; 6%
Veillonella spp.	47; 2%	45; 13%
Clostridium spp.	106; 5%	98; 29%
Propionibacterium spp.	14; 1%	14; 4%
Other anaerobes	10; <1%	10; 3%
Candida spp.	188; 8%	155; 45%
Candida albicans	84; 4%	84; 24%
Candida tropicalis	17; 1%	17; 5%
Candida glabrata	51; 2%	51; 15%
Candida krusei	3; <1%	3; 1%
Candida norvegensis	1; <1%	1; <1%
Candida dublinensis	2; <1%	2; 1%
Candida robusta	1; <1%	1; <1%
other candida spp.	29; 1%	28; 8%
Magnusiomyces spp.	1; <1%	1; <1%

*Enterococcus spp.* were the most prevalent microorganisms (504;22%), isolated from 297 of 343 stents; followed by *Klebsiella spp.* (218;10%), *Streptococcus spp.* (213;9%), *Candida spp.* (188;8%) and *Escherichia spp.* (182;8%), which were cultivated from 180(52%), 170(50%), 155(45%) and 170(50%) stents, respectively. 409(18%) anaerobic bacteria were found on 212(62%) stents. 149(7%) *Prevotella spp.* were isolated from 123(36%) stents, representing the most common anaerobic microorganisms. *Clostridium spp.* (106; 5%) were the second, *Bacteroides spp. (64; 3%)* the third most isolated anaerobic bacteria, cultured from 98(29%) and 50(15%) stents, respectively. *Candida spp. grew* on 155(45%) biliary stents. *Candida albicans* (84; 4%) was the most prevalent *Candida spp.*, followed by *Candida glabrata* (51; 2%).

Analysis of spectrum of microorganisms (Table 3) in relation to an indwelling time of 1 d–15 d (group 1), 20 d–59 d (group 2) and 60 d–3087 d (group 3) revealed that stents extracted after a indwelling period of 1 d–15 d were mainly colonized by aerobic gram-positive bacteria (43/49;88%) and *Candida spp.* (25/49;51%). Aerobic gram-negative bacteria and anaerobes had a proportion of 45%(22/49) and 24%(12/49), respectively. In contrast, stents extracted after an indwelling time of 60 d–3087 d showed an almost equal colonization rate by aerobic gram-negative (176/184;96%) and aerobic gram-positive bacteria (183/184;99%) and a high proportion of anaerobes (127/184;69%). Among aerobic gram-positive bacteria [P<0.01; 88% (group 1), 98% (group 2), 99% (group 3)], the incidence of *Enterococcus spp.* significantly changed with the indwelling time [P<0.01; 49% (group 1), 90% (group 2), 95% (group 3)]. In addition, the colonization rate of anaerobes [P<0.01; 24% (group 1), 67% (group 2), 69% (group 3)] and aerobic gram-negative microorganisms [P<0.01; 45% (group 1), 90% (group 2), 96% (group 3)], in particular the family of Enterobacteriaceae [P<0.01; 35% (group 1), 82% (group 2), 93% (group 3)] altered with the indwelling time.

**Table 3:** The spectrum and the number of microorganisms in relation to the indwelling time of the stents.

	Number of microorganisms isolated from stents, with an indwelling time (1–15)d	Number of microorganisms isolated from stents, with an indwelling time (20–59)d	Number of microorganisms isolated from stents, with an indwelling time (60–3087)d	Number of stents isolated from, with an indwelling time (1–15)d	Number of stents isolated from, with an indwelling time (20–59)d	Number of stents isolated from, with an indwelling time (60–3087)d	P-Value
Number of stents*				49	100	184	
Total number (microorganisms)	178	660	1386				
Median number (microorganisms) (range)	3(0–10)	6(2–13)	8(2–14)				
Aerobic gram-positive bacteria	92; 52%	277; 42%	524; 38%	43; 88%	98; 98%	183; 99%	P<0.01
Enterococcus spp.	28; 16%	162; 25%	300; 22%	24; 49%	90; 90%	175; 95%	P<0.01
Enterococcus faecium	16; 9%	46; 7%	61; 4%	16; 33%	46; 46%	61; 33%	P = 0.08
Enterococcus faecalis	6; 3%	69; 10%	142; 10%	6; 12%	69; 69%	142; 77%	P<0.01
Other Enterococcus spp.	6; 3%	47; 7%	97; 7%	6; 12%	37; 37%	85; 46%	P<0.01
Streptococcus spp.	29; 16%	59; 9%	119; 9%	21; 43%	47; 47%	98; 53%	P = 0.34
Streptococcus viridans group	29; 16%	58; 9%	118; 9%	21; 43%	46; 46%	98; 53%	P = 0.30
Streptococcus (Beta-haemolytic)	0	1; <1%	1; <1%	0	1; 1%	1; 1%	P = 1.00
Staphylococcus spp.	12; 7%	8; 1%	18; 1%	10; 20%	8; 8%	18; 10%	P = 0.06
Staphylococcus aureus	1; 1%	3; <1%	7; 1%	1; 2%	3; 3%	7; 4%	P = 1.00
Staphylococcus coagulase-negative	11; 6%	5; 1%	11; 1%	9; 18%	5; 5%	11; 6%	P = 0.02
Other aerobic gram-positive bacteria	23; 13%	48; 7%	87; 6%	18; 37%	32; 32%	67; 36%	-
Aerobic gram-negative bacteria	35; 20%	204; 31%	511; 37%	22; 45%	90; 90%	176; 96%	P<0.01
Enterobacteriaceae	26; 15%	165; 25%	453; 33%	17; 35%	82; 82%	171; 93%	P<0.01
Escherichia spp.	8; 4%	44; 7%	122; 9%	8; 16%	43; 43%	112; 61%	P<0.01
Klebsiella spp.	5; 3%	54; 8%	154; 11%	4; 8%	48; 48%	123; 67%	P<0.01
Citrobacter spp.	2; 1%	23; 3%	51; 4%	2; 4%	18; 18%	40; 22%	P = 0.02
Proteus spp.	0(0)	8; 1%	29; 2%	0	8; 8%	29; 16%	P<0.01
Morganella spp.	0(0)	4; 1%	13; 1%	0	4; 4%	13; 7%	P = 0.12
Enterobacter spp.	11; 6%	27; 4%	69; 5%	8; 16%	23; 23%	62; 34%	P = 0.02
Serratia spp.	0(0)	0(0)	1; <1%	0	0	1; 1%	P = 1.00
Other Enterobacteriaceae	0(0)	5; 1%	14; 1%	0	5; 5%	14; 8%	-
Non-fermenting bacteria	4; 2%	15; 2%	17; 1%	4: 8%	15; 15%	17; 9%	P = 0.27
Pseudomonas spp.	1; 1%	13; 2%	15; 1%	1; 2%	13; 13%	15; 8%	P = 0.07
Stenotrophomonas spp.	0(0)	2; <1%	1; <1%	0	2; 2%	1; 1%	P = 0.56
Acinetobacter spp.	2; 1%	0(0)	1; <1%	2; 4%	0	1; 1%	P = 0.08
Other non-fermenting bacteria	1; 1%	0(0)	0	1; 2%	0	0	-
Other aerobic gram-negative bacteria	5; 3%	24; 4%	41; 3%	3; 6%	20; 20%	35; 19%	-
Anaerobes	18; 10%	125; 19%	254; 18%	12; 24%	67; 67%	127; 69%	P<0.01
Bacteroides spp.	2; 1%	18; 3%	44; 3%	2; 4%	13; 13%	35; 19%	P = 0.03
Prevotella spp.	10; 6%	48; 7%	87; 6%	8; 16%	39; 39%	73; 40%	P = 0.01
Fusobacterium spp.	0(0)	2; <1%	15; 1%	0	2; 2%	15; 8%	P = 0.02
Veillonella spp.	5; 3%	21; 3%	18; 1%	5; 10%	21; 21%	17; 9%	P = 0.08
Clostridium spp.	1; 1%	28; 4%	75; 5%	1; 2%	26; 26%	69; 38%	P<0.01
Propionibacterium spp.	0(0)	5; 1%	8; 1%	0	5; 5%	8; 4%	P = 0.30
Other anaerobes	0(0)	3; <1%	7; 1%	0	3; 3%	7; 4%	-
Candida spp.	33; 19%	54; 8%	96; 7%	25; 51%	42; 42%	83; 45%	P = 0.58
Candida albicans	17; 10%	27; 4%	36; 3%	17; 35%	27; 27%	36; 20%	P = 0.62
Candida tropicalis	1; 1%	7; 1%	9; 1%	1; 2%	7; 7%	9; 5%	P = 0.48
Candida glabrata	5; 3%	12; 2%	33; 2%	5; 10%	12; 12%	33; 18%	P = 0.24
Candida krusei	0	2; <1%	1; <1%	0	2; 2%	1; 1%	P = 0.56
Candida norvegensis	1; 1%	0	0	1; 2%	0	0	P = 0.15
Candida dublinensis	0	1; <1%	1; <1%	0	1; 1%	1; 1%	P = 1.00
Candida robusta	0	1; <1%	0	0	1; 1%	0	P = 0.45
Other Candida spp.	9; 5%	4; 1%	16; 1%	8; 16%	4; 4%	16; 9%	-
Magnusiomyces	0	0	1; <1%	0	0	1; 1%	P = 1.00

*8 stents could not be included to this analysis due to an unknown indwelling time.

Table 4 illustrates the analysis of the spectrum of microorganisms comparing stents with and without sludge. To avoid confounding by the indwelling time, only stents with an indwelling time ≥60 days (group 3) were included to the stents. In the univariate analysis, significantly more stents with sludge were colonized by *Streptococcus spp.* [P = 0.02; 60(61%) vs. 38(44%)], *Staphylococcus spp*. [P = 0.01; 15(15%) vs. 3(3%)], *Bacteroides spp*. [P = 0.04; 24(24%) vs. 11(13%)], *Prevotella spp*. [P = 0.03; 47(48%) vs. 27(31%)], *Fusobacterium spp*. [P = 0.03; 12(12%) vs. 3(3%)] and *Clostridium spp*. [P = 0.02; 47(48%) vs. 22(26%)], compared to stents without sludge. All microorganisms with a significant difference in colonization rates between stents with and without sludge in the univariate analysis were analyzed in a multivariate logistic regression model. According to multivariate analysis, there was only a significant relationship between the occurrence of sludge and the isolation of *Clostridium spp*. [(P = 0.02; OR (95% CI): 2.4(1.1–4.9)] and *Staphylococcus spp*. [(P = 0.03; OR (95% CI): 4.3(1.1–16.5)].

**Table 4:** Comparison of the spectrum of microorganisms between stents with and without sludge.

	Sludge: Number of microorganisms isolated from stents	No Sludge: Number of microorganisms isolated from stents	Sludge: Number of stents isolated from	No Sludge: Number of stents, Isolated from	UnivariateAnalysis	MultivariateAnalysisP-ValueOdds ratio (95% CI)
Number of microorganisms	831(100%)	555(100%)	-	-	-	-
Number of stents	-	-	98(100%)	86(100%)	-	-
Median indwelling time in days	93	93	93	93	-	-
						-
Aerobic gram-positive bacteria	317; 38%	207; 37%	98; 100%	85; 99%	P = 0.47	-
Enterococcus spp.	176; 21%	124; 22%	95; 97%	80; 93%	P = 0.30	-
Enterococcus faecium	34; 4%	27; 5%	34; 35%	27; 31%	P = 0.64	-
Enterococcus faecalis	78; 9%	64; 12%	78; 80%	64; 74%	P = 0.40	-
Other Enterococcus spp.	64; 8%	33; 6%	55; 56%	30; 35%	-	-
						-
Streptococcus spp.	74; 9%	45; 8%	60; 61%	38; 44%	P = 0.02	P = 0.16; 1.6(0.8–3.1)
Streptococcus viridans group	74; 9%	44; 8%	60; 61%	38; 44%	P = 0.02	-
Streptococcus (Beta-haemolytic)	0	1; <1%	0	1; 1%	P = 0.47	-
Staphylococcus spp.	15; 2%	3; 1%	15; 15%	3; 3%	P = 0.01	P = 0.03; 4.3(1.1–16.5)
Staphylococcus aureus	7; 1%	0	7; 7%	0	P = 0.02	-
Staphylococcus (coagulase-neg.)	8; 1%	3; 1%	8; 8%	3; 3%	P = 0.18	-
Other aerobic gram-positive bacteria	52; 6%	35; 6%	40; 41%	27; 31%	-	-
Aerobic gram-negative bacteria	302; 36%	209; 38%	95; 97%	81; 94%	P = 0.48	-
Enterobacteriaceae	260; 31%	193; 35%	93; 95%	78; 91%	P = 0.27	-
Escherichia spp.	69; 8%	53; 10%	65; 66%	47; 55%	P = 0.11	-
Klebsiella spp.	91; 11%	63; 11%	70; 71%	53; 62%	P = 0.16	-
Citrobacter spp.	29; 3%	22; 4%	25; 26%	15; 17%	P = 0.19	-
Proteus spp.	16; 2%	13; 2%	16; 16%	13; 15%	P = 0.82	-
Morganella spp.	5; 1%	8; 1%	5; 5%	8; 9%	P = 0.27	-
Enterobacter spp.	41; 5%	28; 5%	36; 37%	26; 30%	P = 0.35	-
Serratia spp.	1; <1%	0	1; 1%	0	P = 1.00	-
Other Enterobacteriaceae	8; 1%	6; 1%	8; 8%	6; 7%	-	-
Non-fermenting bacteria	10; 1%	7; 1%	10; 10%	7; 8%	P = 0.63	-
Pseudomonas spp.	8; 1%	7; 1%	8; 8%	7; 8%	P = 1.00	-
Stenotrophomonas spp.	1; <1%	0	1; 1%	0	P = 1.00	-
Acinetobacter spp.	1; <1%	0	1; 1%	0	P = 1.00	-
Other non-fermenting bacteria	0	0	0	0	-	-
Other aerobic gram-negative bacteria	32; 4%	9; 2%	27; 28%	8; 9%	-	-
						-
Anaerobes	167; 20%	87; 16%	79; 81%	48; 56%	P<0.01	-
Bacteroides spp.	30; 4%	14; 3%	24; 24%	11; 13%	P = 0.04	P = 0.83; 2.2(0.9–5.3)
Prevotella spp.	57; 7%	30; 5%	47; 48%	27; 31%	P = 0.03	P = 0.44; 1.3(0.6–2.8)
Fusobacterium spp.	12; 1%	3; 1%	12; 12%	3; 3%	P = 0.03	P = 0.24; 2.3(0.5–9.2)
Veillonella spp.	11; 1%	7; 1%	10; 10%	7; 8%	P = 0.14	
Clostridium spp.	49; 6%	26; 5%	47; 48%	22; 26%	P = 0.02	P = 0.02; 2.4(1.1–4.9)
Propionibacterium spp.	7; 1%	1; <1%	7; 7%	1; 1%	P = 0.07	-
Other Anaerobes	1; <1%	6; 1%	1; 1%	6; 7%	-	-
Candida spp.	44; 5%	52; 9%	39; 40%	44; 51%	P = 0.12	-
						-
Candida albicans	21; 3%	15; 3%	21; 21%	15; 17%	P = 0.50	-
Candida tropicalis	6; 1%	3; 1%	6; 6%	3; 3%	P = 0.51	-
Candida glabrata	10; 1%	23; 4%	10; 10%	23; 27%	P = 0.04	P = 0.10; 0.5(0.2–1.2)
Candida krusei	1; <1%	0	1; 1%	0	P = 1.00	-
Candida robusta	0	0	0	0	-	-
Candida dublinensis	0	1; <1%	0	1; 1%	P = 0.47	-
Other Candida spp.	6; 1%	10; 2%	6; 6%	10; 12%	-	-
Magnusiomyces	1; <1%	0	1; 1%	0	P = 1.00	-

The relationship between the number of microorganisms, sideholes, the diameter as well as the indwelling time of the stent and the occurrence of sludge was also analyzed by multivariate logistic regression analysis. There was a significant relationship between the number of microorganisms [P<0.01; OR (95% CI): 1.3(1.1–1.5)], the indwelling time [P<0.01; 1–15 d vs. 20–59 d: OR (95% CI): 5.6(1.4–22), 1–15 d vs. 60–3087 d: OR (95% CI): 9.5(2.5–35.7)], the presence of sideholes [P<0.01; OR (95% CI): 3.5(1.6–7.9)] and the occurrence of sludge, whereas no significant association between sludge and the diameter of the stent was found [P = 0.32; OR (95% CI): 1.1(0.8–1.5)].

Stent occlusion occurred in 70/343(20%) stents after a median indwelling time of 70 days. In 35% (19/54) of cases, stent occlusion resulted in cholangitis or cholestasis.

## Discussion

Previous analyses of the spectrum of microorganisms isolated from explanted biliary stents were performed with a small series of stents [17], [18]. The strength of this study lies in the large sample size and the sonication method, allowing a better release of pathogens from the biofilm on the surface of the stent [19]–[21]. In the current study, 2283 microorganisms isolated from 343 stents were included into the analysis. The incidence of anaerobes (18%) was high, implying that the preanalytic conditions were correct and preparation of the stents was adequately performed. In literature, the proportion of anaerobes, isolated either from bile or directly from biliary stents ranges between 6% and 40% [16], [22]–[24] and *Bacteroides spp.* are reported to be most frequently isolated biliary anaerobes [16], [23], [25]–[27], which poses a contrast to the present study: *Bacteroides spp.* were only the third most frequently isolated anaerobic microorganisms after *Prevotella spp. and Clostridium spp.*. The reason for our discrepant findings compared to literature may be that identification of microorganisms was performed by matrix-associated laser desorption/ionization-time of flight mass spectrometer (MALDI-TOF), allowing a more precise differentiation of microorganisms compared to other microbiological testing methods. However, some *Bacteroides spp*. were re-classified as *Prevotella spp.* [28]. The incidence of anaerobes was associated with the presence of sludge which can be attributed to ideal anaerobic conditions inside the sludge. Another explanation is that anaerobic bacteria may have a direct impact on the sludge formation. Thus, *Clostridium spp.* were significantly more frequently detected on stents in the presence of sludge. Bacterial enzymes such as beta-glucuronidase and phosphlipase C play an important role in sludge formation [29]: Bilirubin is deconjungated by beta-glucuronidase and precipates as calcium bilirubinate. Furthermore, phospholipase C can hydrolyse lecithin precipating as calcium palmitate. Both enzymes are produced by *Clostridium spp.*. The high proportion of *Clostridium spp.* in occluded stents might also be of clinical relevance, as stent occlusion results in cholangitis. Infections with *Clostridium perfringens* manifest through a broad variety of clinical course ranging from mild to severe septic courses and the most common origin of septicaemia with *Clostridium perfringens* is the hepatobiliary system [30]. Apart from *Clostridium spp., Staphylococcus spp*. were also significantly more often isolated from stents with sludge, compared to stents without sludge. Similar to our finding, Di Rosa et al. [31] also reported a higher proportion of *Staphylococcus spp.* in occluded stents compared to non-occluded stents. However, we can only hypothesize that the change in biliary spectrum observed in occluded stents may be of clinical relevance in case of acute cholangitis. Most of the stent extractions were performed electively and therefore blood cultures were collected only in a small number of patients presenting acute cholangitis. Consequently, no analysis could be performed to find out whether those microorganisms isolated from blood cultures were similar to the microorganisms isolated from the biofilm on the occluded stents. Furthermore, we can only assume that the microorganisms isolated from the biofilm on the stents surface are similar to the microorganisms in the bile fluid, because no concomitant bile culture was collected. In literature, the clinical relevance of bile collection is controversially discussed. Park et al. [32] analyzed 258 bacteremic cholangitis episodes: Complete agreement with blood cultures was observed in 80 (31%) bile samples, 129 (50%) bile samples showed a partial agreement with the blood culture findings, whereas 49 (19%) bile samples presented completely different microorganisms compared to the blood culture results. Gram-negative bacteria showed a significant higher coincidence rate than gram-positive bacteria. The degree of coincidence between bile and blood culture for *Escherichia coli*, *Klebsiella spp., Enterococcus spp. and Streptococcus spp*. was 71.2%, 53.1%, 34.5% and 26.9%, respectively. There are studies showing a clear association between an indwelling biliary stent and an altered spectrum of pathogens in the bile fluid [16], [33]. In the current series, *Enterococcus spp*. were by far the most prevalent genera. This high proportion of *Enterococcus spp.* is in line with our preceding study [16], revealing that bile collected from patients with stents had a significantly higher incidence of *Enterococcus spp.* compared to bile from patients without a stent. Furthermore, the number of *Enterococcus spp*. isolated from blood cultures was also higher in cholangitis episodes with than without an indwelling stent.

Sludge formation was also significantly associated with an increasing indwelling time and the presence of sideholes. In line with our data, a previous study also reported a higher amount of sludge in polyethylene stents with sideholes in comparison to polyethylene stents without sideholes [34]. An experimental study of biliary endoprosthesis efficiency showed that the presence of multiple sideholes does not increase the efficiency of the endoprosthesis, but impairs its mechanical properties [35]. The authors argued by referring to the law of mechanics of virtue: By increasing the number of sideholes a luminal fluid stream passing through the tube is switched to a micro-disturbed fluid stream resulting in an increased resistance of the stent to the bile flow. In theory, sideholes should guarantee the biliary drainage in case of the proximal and distal main orifice being occluded by plugs or blocked by the wall of the bile duct [34]. However, clinical significance of this hypothesis is still missing [34], [36]. Sung et al. [36] compared the clinical efficacy of stents with and without sideholes in a prospectively randomised control trial. In both stents with and without sideholes, the median time before stent occlusion was similar (7.8 vs. 7.9 weeks). Several studies showed that antimicrobial coatings on medical devices are effective against microbial biofilm formation. According to Agostinho et al. [37] antimicrobially coated materials are able to significantly inhibit bacterial biofilm formation through microorganisms like *Staphylococcus spp*. over a certain time. A recent study [38] reported similar findings, analyzing novel high efficiency coatings on anti-microbial surgical sutures using chlorhexidine in fatty acid slow-release carrier systems. Furthermore, the use of new coatings like diamond like carbons (DLC) seems to be a promising approach to reduce biofilm formation on medical devices. Due to its low friction coefficient, high biocompability, chemical inertness, and both high hardness as well excellent smoothness [39]–[41], diamond like carbon is virtually predestined for use as a coating for biliary stents. Laube et al. [42] investigated the ability of diamond-like carbon to decrease formation of crystalline bacterial biofilm as well as stent related side effects and reported an excellent handling, a less painful replacement procedure and high tolerance of application. Furthermore, no crystalline biofilm formation could be detected on the stent surface in vivo and the number and severity of symptomatic urinary tract infections was decreased.

In the current series, stents occluded after a median indwelling time of 70 days. However, stent occlusion resulted in a cholangitis or cholestasis in only 35% of the cases. This suggests that stent occlusion is not always associated with clinical symptoms.

The study displays the following limitations: Choice and indwelling time of the polyethylene stents inserted in the bile duct were not influenced. Therefore, time point of stent extraction and use of stents were not standardized.

In conclusion, the spectrum of microorganisms colonizing the inner surface of the stent changed with the indwelling time. The occurrence of sludge was associated with a higher isolation rate of *Staphylococcus spp. and Clostridium spp.*, the presence of sideholes and with an increasing indwelling time. Although these factors may support stent occlusion, a great proportion of stent occlusions proceeded clinically asymptomatic.
